# The Influences of Landscape Features on Visitation of Hospital Green Spaces—A Choice Experiment Approach

**DOI:** 10.3390/ijerph14070724

**Published:** 2017-07-05

**Authors:** Kaowen Grace Chang, Hungju Chien

**Affiliations:** 1 Department of Landscape Architecture, National Chiayi University, Chiayi City 600, Taiwan; 2 Commerce Technology Application Research Division, Commerce Development Research Institute, Taipei City 106, Taiwan; zooist@gmail.com

**Keywords:** hospital, landscape, healing environment, built environment wellbeing, site improvement, choice experiment

## Abstract

Studies have suggested that visiting and viewing landscaping at hospitals accelerates patient’s recovery from surgery and help staff’s recovery from mental fatigue. To plan and construct such landscapes, we need to unravel landscape features desirable to different groups so that the space can benefit a wide range of hospital users. Using discrete choice modeling, we developed experimental choice sets to investigate how landscape features influence the visitations of different users in a large regional hospital in Taiwan. The empirical survey provides quantitative estimates of the influence of each landscape feature on four user groups, including patients, caregivers, staff, and neighborhood residents. Our findings suggest that different types of features promote visits from specific user groups. Landscape features facilitating physical activities effectively encourage visits across user groups especially for caregivers and staff. Patients in this study specify a strong need for contact with nature. The nearby community favors the features designed for children’s play and family activities. People across user groups value the features that provide a mitigated microclimate of comfort, such as a shelter. Study implications and limitations are also discussed. Our study provides information essential for creating a better healing environment in a hospital setting.

## 1. Introduction

People who stay in hospitals, whether their role is a patient, caregiver, or health professional, constantly need to face difficult situations and highly-stressful working conditions. Numerous studies have suggested that visiting and viewing landscaping at hospitals accelerate patients’ recovery from surgery and help staff focus on work [1,2,3,4,5]. This benefit also corresponds with many ancient and historical designs of medical-care environments [6,7,8], reflecting that holistic healing depends on collaborations of in- and out-of-building spaces in a hospital complex. Recently, landscaping and gardens at hospitals have increasingly come to be considered as an integral part of a healthcare environment. This attention on the physical and psychological healing effects of landscaping at hospitals has provided us new opportunities and challenges. Thus, how to plan the outdoor environment that people in hospitals value and enjoy is an appropriate topic for humanitarian practice of quality healthcare.

From the perspective of hospital management, constructing a quality landscape also implicates a significant investment and, therefore, the hospital administration and landscape architect expect people to benefit from the environment through their constant use. Thus, identifying users’ needs and understanding their perceptions help determine whether or not the environment can successfully serve its people and purpose. Understanding user characteristics is one prominent task in environment planning and design [9]. While there is a strong assumption that there is a relationship between users’ roles and their preferred features in landscaping at hospitals, little empirical evidence has been established for a scientific link between the two variables.

Recently, there has been increased attention placed on research in the planning and design of healing environments [6,7,8,9,10,11,12,13]. A growing number of healthcare professionals acknowledge that outdoor landscapes can assist the psychological and physical recovery of patients [14,15,16,17], facilitate relaxation and recovery from mental fatigue of caregivers and hospital staff [14,18], and establish good relations with nearby communities by offering local recreation opportunities.

Studies have recommended designing features that provide positive experience to encourage interest, stimulate senses, and arouse curiosity for people in healthcare settings [6,11]. Tangible landscape features, such as plants, water bodies, trails, and playgrounds provide environmental clues for visitors to evaluate the compatibility between their own purposes and what the environment can offer. For example, the presence of trees indicates a chance of contact with nature, a surrounding of comfortable microclimate, and a possible short stop; the appearance of a playground presents the opportunity of play for children; and the existence of a trail specifies an access and hiking opportunity. The process of determining the compatibility between personal demands and the environment leads to motion and action [12,13].

The diversity of feature types identify the setting’s capability of offering multiple functions of physiotherapy and social support, such as gathering, interacting with others, and exercising [19]. For instance, vegetation stimulates both emotional and physical responses, such as reducing stress, restoring attention [19,20,21,22], and revitalizing senses [7,23]. Spatial factors, such as a friendly location and accessibility [12,19,20], a visible view from the indoors, and the creation of an inviting entrance [19,21], ease patients’ anxiety caused by the lack of information in an unfamiliar hospital setting [8,20]. On a micro-scale, people also care whether the place has adequate shade, comfortable seats, and seating variety, such as being fixed or movable, group or solitary, and their proximity to natural features [6,10,19]. While a number of studies have explored the impact of landscape features on people’s behavior, until this study, we have little quantitative information on how hospital users evaluate such features in terms of their decision for visits.

People spend time in hospitals for different reasons [24]; such as to provide professional services, receive medical care, take care of patients, or use facilities. Since these individuals can be hospital staff, patients, caregivers, or nearby community residents, they are likely to face very different psychological and physical situations, as well as having different motives to be in the gardens, green spaces, or other types of outdoor settings at the hospital [24]. As a result, studies have found that hospital staff, patients, or patients’ families have different opinions regarding the healing environment [13]. Researchers also indicated that demographic factors influence the level of sensitivity to landscape features in a clinical setting [9]. Considering user populations, research has noted the need for understanding staff and other stakeholders in addition to patients in hospital settings [13]. Although the user factor has been identified to influence visits, behaviors, and satisfaction [25,26], studies that measure perceptions of user group for landscape planning at hospitals are few in number. The understanding of the responses of different user groups to landscape features can guide hospital management and planners to determine the optimal use of limited resources to promote benefits of health and wellbeing with their design.

Towards this goal, we need to know, first, which landscape features should be included to effectively encourage the visits of different user groups. Secondly, which landscape features should be prioritized based on their relative importance to different user groups must also be determined. To answer these questions, we will use the framework of discrete choice modeling (DCM) [27,28,29] to elicit choices among design alternatives (details in Section 2.3) and develop experimental choice sets to investigate how landscape features link to users’ visitation.

## 2. Research Design and Methods

The main purpose of this study is to assess how the presence of landscape features influences the visit intent of users in different roles in a hospital setting. We developed a discrete choice experiment to provide quantitative evidence that indicates the effect and effect size of the landscape features. The objectives of this study are to, firstly, identify the effective landscape features that engage visits; and, secondly, rank the choices of landscape features specific to different user groups based on the effect on the engagement of visits. This study implicates the relevant inclusion and priorities of landscape features for the diverse user groups on a hospital property.

### 2.1. Study Site

**Figure 1:**
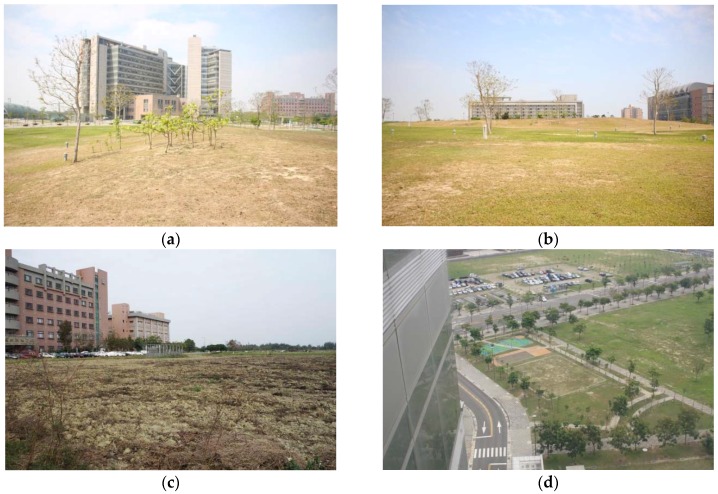
(**a**–**d**) Photos demonstrate the study site surroundings.

The study site is the Chiayi Chang Gung Memorial Hospital, Chiayi County, Taiwan, which is located in a suburban area with a landscape area of nearly 3.3 hm^2^ surrounding the hospital main building (Figure 1). It is located immediately to the boundary between subtropical and tropical climate zones (the Tropic of Cancer). This hospital has approximately 1300 inpatient beds, and employs nearly 1260 staff including medical doctors, nurses, technicians, and staff in management and administrative departments. It is classified as a regional hospital offering comprehensive clinical services including regular treatment, emergency treatment, hospitalization, and long-term care in eight main medical categories including department of medicine, surgery, stomatology, obstetrics and gynecology, pediatrics, traditional medicine, and cancer department. Currently, the hospital has a large ratio of landscape area covered only by lawns. The hospital management has an aim to improve the functionality of its outdoor areas in order for them to become an integral part of the hospital environment and to benefit diverse user groups in and around the hospital.

### 2.2. User Categories

In this study, we distinguished the users into four groups as patients, caregivers, hospital staff, and local residents. The patients were defined as the recipients of health care service in Chiayi Chang Gung Memorial Hospital. Caregivers are people who accompany patients during their treatments in the hospital or take care patients’ daily living in the hospital. They can be patients’ family members or professional caregivers. Hospital staff comprised the personnel hired to work at the hospital either to provide medical services, such as medical doctors and nurses, or the general hospital staff, such as the staff in various management or administrative departments. Local residents were defined as residents who lived within 500 m radius around the hospital. Since patients’ visitors only stay a very short period of time at the hospital, they were not considered in any of the categories.

### 2.3. Discrete Choice Modeling Approach

Discrete choice modeling (DCM) [27,28,29] is utilized as the experiment design in this study. The DCM approach is one of the most promising techniques to elicit the choice behavior from individuals or segments of a population in a particular context [30]. It is a survey-based quantitative technique introduced by Louviere and Hensher (1982) [27]. Generally, in this technique, respondents are asked to choose among defined alternatives that maximize their utility. One advantage of the DCM approach is that researchers are able to present choice alternatives to decision-makers to elicit decision-makers’ preferences in a controlled environment. This technique also has been applied to model choice behavior in other research areas, such as tourism [31,32], transportation [27,33], and environment management [34,35]. Yet, few applications of the DCM technique can be found from the aspects of landscape improvements and valuations [36,37,38,39]. To the best of our knowledge, there is no literature proposing a discrete choice experiment for modeling users’ choices in landscape planning at hospitals. Based on landscape features and their associated levels, this study focuses on modeling the different users’ choice of landscape designs at a hospital and eliciting information on design preferences.

### 2.4. Discrete Choice Experiment and Questionnaire Design

We developed our choice experiments in the following steps. First, we conducted intensive literature reviews and interviews with the hospital administration to define the relevant features and their associated levels in the choice sets. To ensure the relevance, we selected seven features that are feasible to be constructed in the hospital and support either psychological or physiological benefits as suggested in literature [3,6,7,9,12,13,19]. The features may provide opportunities for relaxation, social, low-impact and vigorous activities such as walking, stretching, talking, contacting with nature, running, contemplation, viewing, and playing. As a result, the seven identified features were “tree canopy coverage”, “flower bed areas”, “paved trails”, “water bodies”, “shelters”, “sitting benches”, and “play/fitness equipment”. In this study, lawn was considered as the essential element for all designs because of its general ground cover functionality. Therefore, lawns were not considered as a feature in the design.

The features of tree canopy coverage and flower bed area are each defined as either abundant (100%), moderate (60%), or open (20%) based on their relative ratio of pixels. The level of 0% in tree coverage and flower bed was not included because the current tree coverage was very scarce at the study site, in order to improve from the condition, the level of 0% in tree coverage and flowerbeds was not an option. The associated levels of water body, shelter, sitting benches, paved trail, and play/fitness equipment were set at two levels: presence or non-presence. A description of the seven features and their associated levels in this research are reported in Table 1.

In the second step, we shaped our choice experiments in an attribute-only main effect framework [40] to focus on the principal objective of this study. The attribute-only design presents landscape alternatives consisting of a combination of indicated levels on the identified seven features in a choice set. The seven identified features and their specific levels are shown in Table 1. Through comparisons among choice sets, researchers are able to screen the features for significance and determine which landscape designs are most preferred.

In a full factorial treatment design, there were totally $288(3^{2}\times 2^{5})$ different possible alternatives. In order to optimize the feasible number of survey questions and the model effect, a D-efficiency fractional factorial treatment design [41] was employed and evaluated using the program SAS Version 9.2 (SAS Institute Inc., Cary, NC, USA) [42,43]. As a result, there were 36 landscape alternatives generated. The 36 alternatives were paired and randomly assigned into three questionnaires.

We used the outdoor setting at the Chiayi Chang Gung Hospital as the image background to simulate landscape alternatives and develop visualized material in a photo format. The visualized alternatives shared identical backgrounds that contained elements of buildings, sky, and the lawn area. For the consideration that the general public needs to clearly identify and recognize the functions of features, the feature images used in the simulated photos were chosen based on the common appearances as they were in public green spaces. The image of the background was taken and selected by the researcher. The raw material of the individual features were first selected from the free-of-license image pools and then were edited in the image process software Adobe Photoshop CS6 (Adobe Systems Inc., San Jose, CA, USA) to conduct image extraction, cleaning, perspective change, color adjustment, shadow creation, sunlight cohesion, feature placement, and pixel estimation.

The choice alternatives of landscapes were then graphically presented to the participants in questionnaires. Each questionnaire contained six choice tasks and each task included two design alternatives (i.e., Option 1 and Option 2) and one “I will choose neither of them” option. For each choice task, participants had to state their choice among the three options. In order to reduce the order effect and learning effect, the order of choice tasks in questionnaires were randomized. Along with the responses to the choice experiment, participants were asked to identify their roles according to written descriptions and oral explanations by the surveyors. Participants also answered a set of questions regarding the current involvement with the hospital outdoor space and their demographic information.

**Figure 2:**
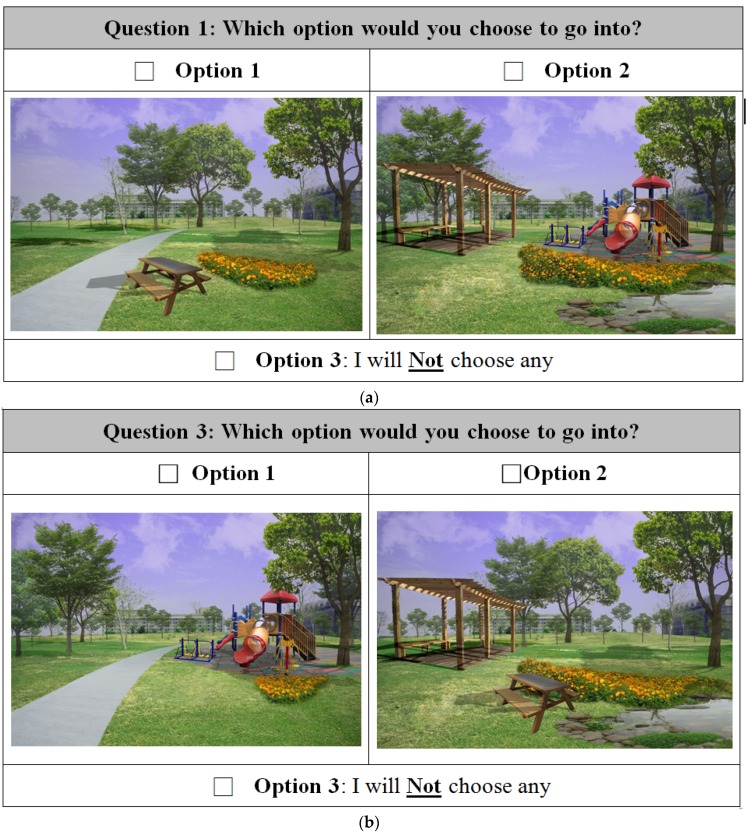
(**a**,**b**) Examples of a choice set in the experiment.

The calculation and statistical examination were performed in the computer program R version 3.2.5 (R Foundation for Statistical Computing, Vienna, Austria) [44] using the multinomial logistic regression technique [45] to analyze the relationship of the respondents’ choices and the characteristics of the landscape designs at the hospital. Two examples of a choice task in choice experiment are presented in Figure 2.

### 2.5. The Estimation Model

The theoretical base of DCM [27,28,29] is random utility maximization theory, introduced by McFadden in 1974 [46], which presumes that every individual makes their decision rationally and that maximizes their utility. To illustrate the basic model behind choice experiment, we consider users’ choice for a landscape from a set of different design alternatives. Let $u_{i}$ be the utility of a concept design and there are *q* available landscape alternatives in the choice set S. Each design includes *m* attributes and each attribute contains a number of levels. Thus, the *q* designs have the utilities $u_{1}\left(X\right)$, $u_{2}\left(X\right)$, …, $u_{q}\left(X\right)$, where *X* is a m×1 vector. The assigned utility to each design depends on the attributes of the design and/or of the characteristics of the user. Then the users choose the design having the greatest utility on that choice occasion; that is, design *i* is chosen in preference to design *j* if and only if:

$$u_{i}\left(X\right)>u_{j}\left(X\right),\;\mathrm{for}\;\mathrm{all}\;j\neq i\in S,$$

where j≠i =1, …, q ∈S. Further, random utility theory assumes that the perceived utility, then, can be expressed with two parts: a systematic utility $v_{j}^{i}$ and a random residual $\varepsilon_{j}^{i}$. The residual $\varepsilon_{j}^{i}$ is distributed independently and identically. The perceived utility is given by the following expression:

(1) $$U_{j}^{i}=V_{j}^{i}+\varepsilon_{j}^{i},\;\forall j\in I,$$

where $V_{j}^{i}$ can be explained by the attributes of the alternatives as well as the characteristics of individuals, and $\varepsilon_{j}^{i}$ captures the combined effects of the various factors affecting the uncertainty, including heterogeneities among individuals or imperfect information. The multinomial logit model is obtained by assuming that each residual, $\varepsilon_{j}^{i}$, is distributed independently and identically.

Based on the Gumbel distribution [47] and type I extreme value distribution [48], the probability density function and cumulative density function of the residual are given as follows:

(2) $$f\left(\varepsilon_{j}^{i}\right)=e^{-\varepsilon_{j}^{i}}e^{-e^{-\varepsilon_{j}^{i}}},$$

(3) $$\mathrm{and}\;F\left(\varepsilon_{j}^{i}\right)=e^{-e^{-\varepsilon_{j}^{i}}}.$$

If $\varepsilon_{j}^{i}$ and $\varepsilon_{k}^{i}$ are the independently and identically distributed extreme value, the distribution of the difference between $\varepsilon_{j}^{i}$ and $\varepsilon_{k}^{i}$ follows logistic distribution. That is, $\varepsilon_{jk}^{i}=\varepsilon_{j}^{i}-\varepsilon_{k}^{i}$ is distributed logistic. The cumulative density function of $\varepsilon_{jk}^{i}$ is given as:

(4) $$F\left(\varepsilon_{jk}^{i}\right)=\frac{e^{\varepsilon_{jk}^{i}}}{1+e^{\varepsilon_{jk}^{i}}}.$$

Since the variation in attributes to the alternatives, the assigned utility to the alternative j by individual i is not identified with certainty to the external observers. Thus, it is not usually possible to predict which alternative will be chosen with certainty for individual i. However, it is possible to express the probability that the individual i chose alternative j conditional on the choice I.

Following McFadden [46], the probability of individual i choosing alternative j is given as the following expression:

(5) $$P^{i}\left(j|I\right)=\mathrm{Pr}\left[\;V_{j}^{i}+\varepsilon_{j}^{i}>V_{k}^{i}+\varepsilon_{k}^{i},\;\forall k\neq j,\;k\in I\;\right]\;\;=\mathrm{Pr}\;[\;\varepsilon_{k}^{i}<\varepsilon_{j}^{i}+V_{j}^{i}-V_{k}^{i},\;\forall k\neq j,\;k\in I\;].$$

The probability of choosing alternative j for individual i depends on systematic utility competing with all alternatives and the joint probability of the random residuals. This probability will rely on the distribution of the random residuals of the systematic utility. Since the residuals are independent and are not given, the choice probability is the integration of $P^{i}\left(j|I\right)$ conditional on $\varepsilon_{k}^{i}$ over all $\varepsilon_{k}^{i}$ and weighted by its own cumulative density. Thus, the choice probability with a close form as follows:

(6) $$P^{i}\left(j|I\right)=\frac{\exp\left(v_{j}^{i}|\theta \right)}{\sum_{k=1}^{m}\left(v_{k}^{i}|\theta \right)},\;k\in I.$$

Decision-maker’s utility is usually linearly specified in parameters, that is $V_{j}^{i}=\beta^{\prime }x_{j}^{i}$, where $x_{j}^{i}$ is a vector of observed attributes for alternative j and/or the characteristics of the individual. Therefore, the multinomial/conditional logit probabilities become:

(7) $$P^{i}\left(j|I\right)=\frac{exp^{\beta^{\prime }x_{j}^{i}}}{\sum_{k}exp^{\beta^{\prime }x_{k}^{i}}},\;k\in I.$$

### 2.6. Data Collection Procedure and Participants

Individuals were recruited at six spots at the outdoor areas at Chiayi Chang Gung Memorial Hospital and two public open spaces within 200 m distance to the hospital for voluntary and anonymous participation. Among the six spots at the hospital, four spots were immediate to the front, rear, east, and west entrances of the hospital main building where roof shades and seating were offered and another two spots were at the areas near the front entrance where trees had grown and shaded seating was provided. The two spots outside the hospital offered grown trees and shaded seating. When the trained surveyors arrived at the site, they wore the vests provided for hospital workers and selectively approached potential respondents who appeared to be either not rushed for any task, sitting and relaxed, leisurely doing some activities, such as strolling or playing, or enjoying viewing other people or landscapes. After participants had agreed to join the experiments, face-to-face interviews were conducted on site to fill out the experimental survey. The time a respondent took for an interview approximately ranged from 8–15 min. The data collection time was from 7 am to 12 pm and 1 pm to 6 pm for seven days, including five weekdays and two weekend days.

At the end, 419 out of 432 interviewed respondents validly completed the survey, which consists of six choice tasks, producing a total of 2514 choice experiments. Participants were grouped into four categories based on their roles and tasks in the hospital, including patients (16.71%), caregivers (38.42%), hospital staff (31.50%), and local residents (13.60%). The socio-demographics of the respondents are shown in Table 2. The distribution between male and female respondents is fairly even (48.93% and 51.07%, respectively). Approximately one third of the respondents’ ages are between 30 and 39 years old (30.75%), followed by the age of 40–49 (21.24%), 50–59 years old (17.42%), 20–29 years old (15.27%), greater than 60 years old (10.98%), and 18–20 years old (4.3%). The majority of participants are college-educated (44.94%) or less (49.28%).

In the current state, the use of the hospital outdoor area is reported in Table 3. The differences among user groups are mainly in the use time, duration, and the number of companions. On average, approximately 45% of the respondents visit the hospital outdoor area one to two times per week. The most popular time segments are 8:00 am to 11:00 am 4:00 pm to 7:00 pm. About half of the respondents stayed in the outdoor space less than 30 min, and one third would stay 30 min to 1 h. The patients prefer to be in the outdoor areas in the morning, while the caregivers use the outdoor area more evenly, and staff and residents are more active in the evening. In comparison, the residents are shown to spend longer time in the area and have a greater number of companions.

## 3. Results

In this section, we report the findings of whether, and to what extent, the landscape features affect the visitation to landscaping at the hospital. To answer these questions, we measured participants’ choices in various landscape alternatives by using the experimental framework of discrete choice modeling [27,28,29] and D-efficiency fractional factorial treatment [41] in SAS V.9.2 (SAS Institute Inc., Cary, NC, USA) [42], and analyzed the data using a multinomial logit regression technique in R V.3.2.5 (R Foundation for Statistical Computing, Vienna, Austria) [44,45] as described in Research Design and Methods.

### 3.1. Feature Effectiveness on Visit Intention

In this section, we report which landscape features affect the visits of different user groups. To answer this question, we conducted five multinomial logit models that included a base model that estimates the effects of pooled users and four models that estimate the effects of individual user groups among patients, caregivers, hospital staff, and local residents. The features included the designated combinations of “tree canopy coverage”, “flower bed area”, “paved trail”, “water body”, “shelter”, “sitting bench”, and “play/fitness equipment” in different alternative levels.

Table 4 shows the estimated results for the pooled sample of respondents. We estimate the multinomial logit regression model [45] grounded on data obtained from the choice-based questionnaires with the statistic software R (R Foundation for Statistical Computing, Vienna, Austria) [44]. First, the likelihood ratio chi-square test shows the model fit is significant at $x^{2}=239.02$ and p<0.001, which indicates our full model is significantly better than the null model. The intercept terms are noted as the alternative specific constant (ASC) in multinomial logit models. The estimated coefficients for the two intercept terms are statistically significantly different from zero with positive values, which confirms the landscape features positively influence respondents’ visits (Table 4).

For pooled responses, the findings representing the general hospital users show the coefficients of features of “tree canopy coverage”, “flower bed areas”, “trails”, “shelters”, “sitting benches”, and “play/fitness equipment” are positively and significantly different from zero, indicating landscapes at hospitals which include those features create a more attractive environment for the general users, while the feature “water body” shows no effect on visits. The magnitude of coefficients in Table 4 shows their relative influences in prediction. We found the feature of “shelters” has the greatest influence on visit among all features, the second one is “play/fitness equipment”, the third one is “trails”, and the last one is “flowerbed areas”.

Table 5 shows the estimated results of the multinomial logit models with the choices, respectively, among the four user groups. In the model of patients, we found the features of “tree canopy coverage”, “trails”, “water bodies”, “shelters”, and “sitting benches” significantly influence the intent of visiting the outdoor area. The positive signs of these features are as we expected. Among all significant features, the most influential feature affecting the patients’ willingness of entering the outdoor space is “shelter”, followed by “sitting benches”, “tree canopy coverage”, “trails”, and “water bodies”.

In comparison among the findings of each user group, we found caregivers and hospital staff show rather similar preferences. The features of “trails”, “shelters”, “sitting benches”, and “play/fitness equipment” are significant with positive signs while the “water bodies” is negatively significant for hospital staff. Among features, the “trails”, “shelters”, and “play/fitness equipment” can be considered the most influential features for caregivers and hospital staff, respectively. To the local residents, they value fewer features compared to other user groups. The features of “shelters” and “play/fitness equipment” are the two significant features encouraging their visits to a hospital outdoor space.

### 3.2. The Odds Ratios of Landscape Features on Visit Intention

In this section, we reported the odds ratio of each landscape feature on each user group (Table 6). The odds ratio is used to determine the probability of visitation when the feature status changes. The predicted probability indicates the degree of impact caused by the change of feature status. With the associated features, the odds ratio is a relative measure of the effect that can be calculated as the exponential of the estimated feature coefficients (*EXP(B)*). In general, the features with positive effects on the logit will display *EXP(B) > 1*, features with no effect on the logit will display *EXP(B) = 1.0*, and features with negative effects on the logit will display *EXP(B) < 1*.

For pooled users, the result shows the feature “shelters” has the largest magnitude in odds ratio among all features (OR = 1.471), which indicates one unit increase in “shelter” would increase 47.1% in odds for respondents to visit the landscape. Secondly, one unit increase in “fitness/play equipment” would increase 46.5% in odds for respondents to use the space (OR = 1.465). “Trails” and “benches” are the third and fourth effective features and their unit odd effects are, respectively, 37.7% and 18.1% increases. The effect of “flowerbed” is relatively smaller (OR = 1.37).

In the patient model, the most influential feature “shelters” shows an increase of 61.4% in odds for patients to enter the outdoor space following one unit change. Consequently, the features of “sitting benches”, “tree canopy coverage”, “paved trails”, and “water bodies” would improve 44.1%, 43.6%, 38.1%, and 22.4% in odds, respectively. In the category of caregivers, the models show the most valued feature, “paved trails”, improve 53.4%, while features of “shelters” and “play/fitness equipment” encourage 53.1% and 39.8% increases in the odds, respectively, for caregivers’ access.

For the people working in the hospital, the landscape design including “play/fitness equipment” would raise the odds of their visits by 61.9%. The second and third effective features “shelters” and “paved trails” would grow the visiting odds by 41.6% and 30.2%. However, the placement of “water bodies” would decrease the odds of hospital staff’s willingness to access the landscape by 17.0%. In comparison with the other groups, fewer features show an influence on local residents’ visits. Between the features, “play/fitness equipment” indicates the ability to encourage an 85.9% increase and “shelter” promotes a 32.8% increase for attracting nearby residents to visit the space.

## 4. Discussion

Contemporary healthcare administration and landscape architects need to know which features are most desired and by whom so that they can best allocate resources and match needs to establish environmental/outdoor settings that are beneficial to users. People that stay or work in hospitals are often under a lot of emotional and work-related stress. Until the findings of this study, we did not have quantitative evidence to know how and to what extent landscape features affect the visitation of various user groups in hospitals. This study investigated the questions as follows: which are the important landscape features that affect people’s intent to visit landscapes in hospital? To what extents do the landscape features affect people’s intent? We used discrete choice modeling in the experimental design and collected empirical data in hospital outdoor settings to estimate the importance of each feature to hospital user groups.

We found that types of features offered in landscaping at the hospital do influence users’ visitation intents. Among landscape features, shelter and play/fitness equipment are most effective for promoting visits to hospital outdoor spaces across user groups. Secondly, the placement of trails and sitting benches can generally encourage people to access the outdoor spaces in a hospital setting. However, flowerbeds and water bodies are relatively less effective, while the performance of tree canopy coverage is inconsistent across different user groups.

The results also present the user-centered ranks of designing features to provide empirical evidence and also practical information for project developments in hospital planning and design. The findings of our study suggest that the type of landscape features in hospital outdoor settings have different powers to promote visits from each group. The four hospital user groups, respectively, value a unique rank of landscape features.

We found that the features of shelter, tree coverage, and bench seats are important to the patient group, in general. This result may link to patients’ physical states and their need for low-impact activities. The significance of tree canopy and water features may indicate patients’ specific needs to relax and enjoy contact with nature. Among features, the existence of shelter greatly increases the chances of patients’ visit. This result may specify the importance of features that can offer patients a sense of control and a comfortable, shaded place that accommodates resting, staying, or social interactions. While the presence of water features promotes patients’ visit, it does not have an effect on caregivers and local residents and has a negative effect to staff. These findings correspond with the dissimilar psychological and physiological states of patients from other user groups. It is also possible that the inclusion of water bodies can imply increasing risks in safety and liability for management and medical staff. As a result, those safety and maintenance concerns may dominate the hospital staff’s decision choice in terms of having a pond-like water feature at work.

With slight differences in effective powers, caregivers and hospital staff have comparable choices in their top three priorities—trail, play/fitness equipment, and shelter. As both roles carry working tasks, the choices of caregivers and hospital staff indicate an expectation that the landscape facilitates opportunities for physical activities and stopping places for restoring and refreshing from their working environment and routine. In comparison with other groups, the local residents greatly value the features of ‘shelters’ and ‘play/fitness equipment’ in the design. This finding suggests the features that can support family-oriented activities and provide nearby recreation would gain a greater chance of residents’ engagement.

Creating a therapeutic and restorative hospital outdoor setting where people regain physical and psychological health is a crucial task. Here, we make planning and design recommendations as follows. First, in addition to essential planting plans, hospital planners and landscape architects should establish opportunities for people to conduct exercise because, in our study, the landscape features that facilitate physical activities effectively encourage more visits in general. Designers can consider including features that assist both mild and vigorous levels of exercise for people in various physiological states in hospital [49]. Specifically, in the areas planning to serve people who work in, and reside near, hospitals, designers can consider features associated with middle- to vigorous levels of physical activities such as sports facilities and fitness equipment, while the areas serving patients can be installed with features that support low-impact and moderate activities with standards of universal design to accommodate friendly access and smooth movements.

Second, in the study, the shelter feature is highly valued across hospital user groups. Moreover, it allows a mitigated microclimate without exposing visitors to hot and cold weather when doing both active and low-impact activities, such as contemplation, viewing, talking, or preparing for activities. Designers and planners should be especially attentive to the quantity and distribution of the placement of shelters.

Third, as patients in this study show a stronger need of nature, hospital management and designers should invest in, and include more, diverse natural elements, such as plants and water features, to emphasize the possible contact with nature in the area targeting on serving patients. This finding and recommendation also echoes with the therapeutic function of natural elements in a number of studies [6,12,13,50].

This study contains some limitations. We presented the landscape features by aiming only at their functionalities. The feature appearances were selected by the principles of being identifiable and recognizable to the general public. However, the look of the features can be a factor that influences people’s choice by their subjective judgment of aesthetics in the experiment. Future studies should also consider the aesthetic characteristics to hospital users’ responses. We conducted the experiment at only one hospital in which there are specific site conditions that include the regional weather characteristics (tropical/subtropical climate), the population composition, and land use of its nearby (suburban residential areas). In addition, this hospital has relatively large landscaping areas and is classified as a regional hospital with comprehensive services provided. The users’ responses in this study can be a specific reflection of those conditions.

We interviewed people in outdoor settings at locations either shaded by trees or roofed structures in different times during the day. Those locations were still varied with regard to temperature and sunlight. Future studies should manage those weather variations in the laboratory to separate its possible impacts. We selected a limited number of features that are feasible to install at the hospital and contributive to health and wellbeing in the literature relating to healing environments. The importance of each feature was ranked individually, and a joint effect of feature combinations was not taken into account in the analyses. Future research may include layout factors, such as adjacency, area partition, relative location, and distance, in study variables so that we obtain a further understanding of how these interconnections affect people’s use of landscaping at a hospital.

## 5. Conclusions

Users of hospital outdoor spaces gain psychological and physical wellbeing and healing benefits through their personal contacts with hospital surroundings in various activities. Such activities include strolling, stretching, exercising, viewing other life forms, enjoying the breeze and sunlight, touching the plants, playing, or just having a moment away from busy and intense situations to energize bodies and enlighten minds. When considering landscaping at hospitals for people in various psychological and physical states, hospital administration and designers in collaboration need to understand their characteristics and make the best decisions to create a place that engages people. The findings in this study provide evidence of which landscape features are effective and what their relative importance are based on the user-centered choices that attract visits. Such research findings are essential as landscaping at hospitals have increasingly been seen as important and integral parts of the health care environment.

## Figures and Tables

**Table 1:** Features and associated levels in choice experiments.

Feature/Attributes	Description	Levels
Tree canopy coverage	The relative ratio of canopy coverage	100%, 60%, 20%
Flowerbed area	The relative ratio of flower bed coverage	100%, 60%, 20%
Trail	Presence of trail in the landscape	1 = Yes, 2 = No
Water body	Presence of water body in the landscape	1 = Yes, 2 = No
Shelter	Presence of the shelter in the landscape	1 = Yes, 2 = No
Bench	Presence of the bench in the landscape	1 = Yes, 2 = No
Play/Fitness equipment	Presence of the play and fitness equipment in the landscape	1 = Yes, 2 = No

**Table 2:** Socio-demographic profile of respondents.

Demographics	Percentage	Demographics	Percentage
Gender		Education	
Male	48.93%	High school	49.28%
Female	51.07%	College	44.94%
		Postgraduate	5.78%
Age		Occupation	
18–20	4.30%	Farming/Fishing/Forestry	5.49%
20–29	15.27%	Production/Manufacturing	10.26%
30–39	30.79%	Business	11.22%
40–49	21.24%	Government worker	4.30%
50–59	17.42%	Home maker	10.50%
60–69	7.16%	Free lancer	7.64%
≥70	3.82%	Student	5.97%
		Service industry	28.16%
		None/Retired	6.21%
		Health care	10.26%

**Table 3:** Respondents’ use of the current hospital outdoor area.

User Role	All Users	Patients	Caregiver	Staff	Residents
Frequency					
<1 time per week	10.50%	23.19%	12.42%	3.03%	7.02%
1–2 times per week	44.87%	40.58%	47.83%	46.21%	38.60%
3–4 times per week	23.15%	21.74%	22.36%	24.24%	24.56%
5–6 times per week	10.02%	4.35%	8.07%	13.64%	14.04%
≥7 time per week	11.46%	10.14%	9.32%	12.88%	15.79%
Time segment					
5 am–8 am	8.00%	6.60%	8.26%	10.38%	3.33%
8 am–11 am	23.85%	33.96%	23.55%	17.92%	26.67%
11 am–2 pm	10.77%	13.21%	12.81%	9.91%	4.44%
2 pm–pm	17.38%	18.87%	22.31%	11.79%	15.56%
4 pm–7 pm	25.54%	18.87%	21.49%	31.13%	31.11%
7 pm–10 pm	12.31%	8.49%	9.92%	15.09%	16.67%
After 10 pm	2.15%	0.00%	1.65%	3.77%	2.22%
Duration					
<30 min	49.52%	55.88%	51.90%	47.33%	40.35%
30–60 min	33.09%	27.94%	31.65%	35.88%	36.84%
1–2 h	10.63%	10.29%	12.03%	8.40%	12.28%
2–3 h	3.14%	1.47%	1.27%	4.58%	7.02%
≥3 h	3.62%	4.41%	3.16%	3.82%	3.51%
Number of companions					
0	17.99%	24.64%	17.50%	15.27%	17.54%
1	41.97%	42.03%	38.13%	53.44%	26.32%
2	30.46%	27.54%	33.75%	23.66%	40.35%
≥3	9.59%	5.80%	10.63%	7.63%	15.79%

**Table 4:** The estimates of landscape features on pooled respondents’ visitation intent.

Items	Landscape Features	Std. Error	Pr (>|t|)
Intercept (Option 1)	2.249 ***	0.169	<0.001
Intercept (Option 2)	2.399 ***	0.166	<0.001
Tree canopy coverage	0.177 **	0.081	0.028
Flowerbed areas	0.128 *	0.077	0.096
Paved trails	0.320 ***	0.043	<0.001
Water bodies	−0.048	0.048	0.322
Shelters	0.386 ***	0.048	<0.001
Sitting Benches	0.166 ***	0.043	<0.001
Play/fitness equipment	0.382 ***	0.044	<0.001
Likelihood ratio test	chisq = 239.02		

Note: * Significant at 0.1 level; ** Significant at 0.05 level; *** Significant at 0.01 level.

**Table 5:** The estimates of landscape features on visitation intent of different user groups.

User Role	Patients			Caregivers			Staff			Residents		
	Landscape Features	Std. Error	Pr (>|t|)	Landscape Features	Std. Error	Pr(>|t|)	Landscape Features	Std. Error	Pr(>|t|)	Landscape Features	Std. Error	Pr (>|t|)
Intercept (Option 1)	3.410 ***	0.747	<0.001	2.852 ***	0.340	<0.001	1.592 ***	0.244	<0.001	2.538 ***	0.483	<0.001
Intercept (Option 2)	3.589 ***	0.743	<0.001	2.961 ***	0.338	<0.001	1.805 ***	0.238	<0.001	2.623 ***	0.477	<0.001
Tree canopy coverage	0.362 *	0.197	0.066	0.181	0.131	0.165	0.104	0.147	0.479	0.170	0.221	0.442
Flowerbed areas	0.179	0.193	0.354	0.070	0.124	0.576	0.152	0.139	0.275	0.093	0.211	0.657
Paved trails	0.323 **	0.107	0.003	0.428 ***	0.071	<0.001	0.264 ***	0.078	<0.001	0.186	0.117	0.113
Water bodies	0.202 *	0.119	0.088	−0.034	0.078	0.668	−0.186 **	0.087	0.032	−0.057	0.132	0.665
Shelters	0.479 ***	0.117	<0.001	0.426 ***	0.080	<0.001	0.348 ***	0.087	<0.001	0.284 **	0.129	0.027
Sitting benches	0.365 ***	0.106	<0.001	0.145 **	0.070	0.037	0.151 *	0.077	0.051	0.039	0.116	0.735
Play/fitness equipment	0.117	0.108	0.276	0.335 ***	0.071	<0.001	0.482 ***	0.080	<0.001	0.620 ***	0.119	<0.001
Likelihood ratio test	chisq = 47.973			chisq = 107.92			chisq = 82.395			chisq = 36.935		

Note: * Significant at 0.1 level; ** Significant at 0.05 level; *** Significant at 0.01 level.

**Table 6:** The order and extent of the impact size of landscape features on user groups’ visitation intent.

Group	All Users		Patients		Caregivers		Staffs		Residents	
Order	Features	OR	Features	OR	Features	OR	Features	OR	Features	OR
1	Shelters ***	1.471	Shelters ***	1.614	Trails ***	1.534	Play/fitness equipment ***	1.619	Play/fitness equipment ***	1.859
2	Play/fitness equipment ***	1.465	Tree canopy coverage *	1.436	Shelters ***	1.531	Shelters ***	1.416	Shelters **	1.328
3	Trails ***	1.377	Sitting benches ***	1.441	Play/fitness equipment ***	1.398	Trails ***	1.302	Trails	1.204
4	Tree canopy coverage **	1.194	Trails **	1.381	Sitting benches **	1.156	Sitting benches *	1.163	Tree canopy coverage	1.185
5	Sitting benches ***	1.181	Water bodies *	1.224	Tree canopy coverage	1.198	Water bodies **	0.830	Flowerbed areas	1.097
6	Flowerbed areas *	1.137	Flowerbed areas	1.196	Flowerbed areas	1.073	Flowerbed areas	1.164	Sitting benches	1.040
7	Water bodies	0.953	Play/fitness equipment	1.124	Water bodies	0.967	Tree canopy coverage	1.110	Water bodies	0.945

Note: * Significant at 0.1 level; ** Significant at 0.05 level; *** Significant at 0.01 level.
